# The role of answer content and length when preparing answers to questions

**DOI:** 10.1038/s41598-024-68253-6

**Published:** 2024-07-24

**Authors:** Ruth Elizabeth Corps, Martin J. Pickering

**Affiliations:** 1 Department of Psychology, The University of Sheffield, ICOSS Building, 219 Portobello, Sheffield, S1 4DP UK; 2 Psychology of Language Department, Max Planck Institute for Psycholinguistics, Nijmegen, Netherlands; 3 Department of Psychology, University of Edinburgh, Edinburgh, UK

**Keywords:** Turn-taking, Dialogue, Question-answering, Speech planning, Human behaviour, Psychology

## Abstract

Research suggests that interlocutors manage the timing demands of conversation by preparing what they want to say early. In three experiments, we used a verbal question-answering task to investigate what aspects of their response speakers prepare early. In all three experiments, participants answered more quickly when the critical content (here, *barks*) necessary for answer preparation occurred early (e.g., *Which animal ****barks**** and is also a common household pet*?) rather than late (e.g., *Which animal is a common household pet and also ****barks***?). In the individual experiments, we found no convincing evidence that participants were slower to produce longer answers, consisting of multiple words, than shorter answers, consisting of a single word. There was also no interaction between these two factors. A combined analysis of the first two experiments confirmed this lack of interaction, and demonstrated that participants were faster to answer questions when the critical content was available early rather than late and when the answer was short rather than long. These findings provide tentative evidence for an account in which interlocutors prepare the content of their answer as soon as they can, but sometimes do not prepare its length (and thus form) until they are ready to speak.

## Introduction

Conversation is a puzzle: Formulating an utterance takes at least 600 ms^1^, but interlocutors’ turns are so finely coordinated that there is often little gap between their contributions (around 200 ms;^2^). Most theories agree that interlocutors achieve such timing by predicting what the current speaker is likely to say, so that they can prepare a response early while still comprehending (see e.g.,^3^ for a review). But what aspects of their response do they actually prepare early? In this paper, we report three question-answering experiments investigating whether speakers prepare just the meaning of their answer early, or whether they also prepare the form.

Much research suggests that next speakers prepare some aspects of their response early (e.g.,^4–7^; though see^8^). For example, Bögels, Magyari, and Levinson^9^ found that participants answered questions more quickly when the critical content necessary for preparation (here *007*) was available early (e.g., *Which character, also called 007, appears in the famous movies*?) rather than late (e.g., *Which character from the famous movies is called 007*?), suggesting that interlocutors used turn content (i.e., what the speaker said) to predict the questions’ likely answer and prepare some aspects of their answer early. Given that speakers (largely) compute content (i.e., meaning) first during language production^10^, Bögels et al.’s^9^ study suggests that interlocutors prepare at least some content early.

But how much of their answer do speakers prepare early? One possibility is that speakers complete all stages of formulation (lemma selection, structure building, and word form retrieval;^10^) early, and so they prepare both the content (conceptualisation and lexicalisation) and form of their answer while still comprehending. We refer to this hypothesis as the *early-form account*. Consistent with this possibility, Bögels et al.^9^ found that answer preparation in the early condition was associated with an event-related potential (ERP) effect in the middle frontal and precentral gyri. These brain areas overlap with areas involved in phonological encoding (see e.g.,^1^), suggesting speakers prepared the form of their answers early. However, this ERP effect has also been associated with other processes, such as retrieving information from memory^11^ or monitoring for a cue (such as the end of the question^12^).

In another study, Barthel and Levinson (^13^; Experiment 3) had Dutch speakers view objects (e.g., an apple, a potato, a strawberry, and broccoli) while answering questions in which the critical content (here, *grows on a tree*) was available early (e.g., *Which object grows on a tree and is also edible?*) or late (e.g., *Which object is edible and grows on a tree*?). On 25% of the trials, the target object (here, the apple with the Dutch name *appel*) disappeared at the beginning of response preparation (determined by participants’ gaze towards the target object) and was replaced by a letter string, which participants judged as a word or not. Judgements were slower and more error-prone for a phonologically related word (e.g., *ampel*; traffic light) than for an unrelated word (e.g., *zaag*; saw). This phonological interference effect occurred regardless of whether participants prepared early or late, suggesting they prepared the form of their answers in both instances, which interfered with their comprehension of phonologically related words. Schnur, Costa, and Caramazza (^14^; see also^15^) also found results to support this conclusion in a picture-naming study. Participants produced sentences of varying lengths (short: *the girl walks*; long: *the orange girl walks*) while ignoring auditory distractors that were phonologically related to the verb (*walnut*). They found that participants were quicker to begin speaking in the presence of a related versus an unrelated distractor word, suggesting they prepared the form of their whole utterance (i.e., because the verb was sentence-final). Note, however, that participants were not responding to a previously produced utterance and were instead producing sentences in isolation.

But in both Barthel and Levinson^13^ and Schnur et al.^14^, the pictures may have primed their corresponding lexical concepts^16^, making object names easy to access during preparation. Research suggests that speakers can prepare more of their response early when they are more familiar with the lexical concepts they will produce^17^. As a result, participants may have prepared the form of their answers early simply because the on-screen pictures primed the object names, which made accessing the lexical concept of the target name easy and allowed them to prepare answer form.

An alternative possibility is that speakers prepare the content of their answer early, but prepare (at least part of) its form late. This *late-form account* would be consistent with speech production models that postulate incremental planning^10^. Speakers may postpone form preparation because dual-tasking production and comprehension is cognitively demanding. For example, preparing a response while simultaneously comprehending increases processing load compared to when just preparing^18^, and dual-tasking two linguistic tasks is demanding—specifically, more demanding than when one task is non-linguistic^19^. Furthermore, there is evidence that preparation interferes with concurrent comprehension^20^. This interference will be particularly strong if speakers prepare both the content and the form of their utterances early.

Evidence compatible with the late-form account comes from studies investigating the scope of advance planning. In a study investigating whether more syntactically complex utterances take longer to plan, Ferreira^21^ found that participants who memorised sentences and produced them 500–1000 ms later were quicker to speak when the sentences were short and contained fewer phonological words (e.g., *The river empties into the bay that borders the little town*) than when they were long and contained more phonological words (e.g., *The large and raging river empties into the bay that borders the little town*). It is possible that participants prepared the content of their sentence during memorisation, but the length effect suggests that they had not prepared the form of these sentences—they needed to generate it when asked to articulate. Thus, participants prepared the content of their sentences early, but the form late.

In a picture-naming study, Meyer^22^ found that participants were slower to produce noun-phrase conjunctions (e.g., *the arrow and the bag*) and sentences (e.g., *the arrow is next to the bag*) when they simultaneously heard a distractor word semantically related to the first or the second noun compared to when it was semantically related to neither noun. Additionally, they were faster when the distractor was phonologically related to the first noun, but not the second noun, than when it was phonologically related to neither noun. These findings suggest that participants prepared the content of both nouns, but prepared the form of only the first noun. Finally, Smith and Wheeldon^23^ found that participants were faster to speak when producing sentences such as *The cat and the cap move up* (phonological overlap in the same phrase) but not when producing sentences such as *The cat moves above the cap* (phonological overlap across phrases), again suggesting participants did not prepare the form of their complete utterance. However, these studies did not manipulate the time-course of preparation (i.e., whether they could begin planning a response early or late).

In sum, we do not know what aspects of their answer speakers prepare early. The early-form account predicts that speakers prepare both the content and the form. The late-form account, in contrast, predicts that speakers prepare content early but form late. We tested between these hypotheses in three experiments using a question-answering task. In all experiments, we manipulated the availability of the critical content (here, *barks*) necessary for answer preparation: it was available either early (e.g., *Which animal barks and is also a common household pet*?; see Table 1) so that participants could potential prepare their answer before the question end, or late (e.g., *Which animal is a common household pet and also barks*?) so that they could not. To determine whether participants prepared the complete form of their answers early, we manipulated the length of the to-be-prepared answers, so that they were either short (single word) or long (multi-word) answers. We assume that long answers require more form preparation than short answers^21^. Answers were not scripted, and participants could choose how they responded. However, the questions were designed to induce similar answers across participants (see “Methods“).

**Table 1:** Example stimuli for the four conditions in Experiment 1.

Answer length	Critical content	Question	Expected answer
Short	Early	Which animal **barks** and is also a common household pet?	Dog
	Late	Which animal is a common household pet and also **barks**?	
Long	Early	Which address, home to the **Prime Minister**, is in London?	Ten Downing Street
	Late	Which address in London is also home to the **Prime Minister**?	

The critical content necessary for preparing an answer is highlighted in bold.

The interaction between these two factors is critical for determining what speakers prepare early. If participants prepare both content and form early (the early-form account), then the factors should interact. When the critical content occurs late, participants are unable to prepare their answer and so they should produce a short answer quicker than a long answer. But when the critical content occurs early, participants are able to prepare most (or all) of the content and the form of their answer, and so the length effect should be smaller. If participants prepare the content of their answer early, but not the form (the late-form account), then the length effect should be the same regardless of whether the critical content occurs early or late, because participants will still need to prepare the form of their answer in both conditions. Note that we present the early- and late-form accounts as two alternatives, but it is also possible that different situations elicit different production strategies. We return to this issue in the Discussion.

We first conducted a pilot study (with 42 native English speakers), which showed that participants answered more quickly when the critical content necessary for answer preparation was available early (*M* = 388 ms) rather than late (*M* = 824 ms). But we found no difference in answer times when to-be-prepared answers were short (*M* = 578 ms) rather than long (*M* = 631 ms) and there was no interaction between these two factors. The lack of interaction supports a late-form account. However, this study had at least two limitations. First, we may have failed to find an interaction because there was no effect of Answer Length, which may have been difficult to detect because the difference in the average word length of answers in the short and long conditions was relatively small (*M*difference = 1.26). In the experiments we report, we increased the difference in the average answer length in the short and the long conditions, thus strengthening our Answer Length manipulation. Second, this experiment used a voice-key to record answer onset, and did not record participants’ actual answers. It is thus possible that the voice-key was (generally) inaccurate and was triggered by false response, or that participants produced more incorrect answers in one condition than the other. Given these limitations, we do not use the results of this pilot study to draw conclusions about whether speakers prepare both the content and the form of their utterances early. Instead, we use the results to derive predictions about expected effect sizes so we can compute Bayes Factors for our effects, especially since the early-form account predicts a null interaction.

All of our experiments were administered online. Although much research has found that language comprehension and language learning can be studied online^24,25^, studying speech production is more difficult because it can be harder to get highly accurate measurements of reaction time. Some research has found large variability in the precision of when images or audio are presented to participants^26,27^. But recent research also suggests that although data collection online may be noisier than in the laboratory, with longer tails in the distribution, onset latencies can be measured with good accuracy^28–30^. These studies have replicated key findings in the speech production literature. For example, Fairs and Strijkers^28^ found a word frequency effect both online and in the laboratory. Although latencies collected online were longer, there was no difference in the size of the word frequency effect in the two experiments. Furthermore, Stark et al.^29^ found a cumulative semantic interference effect in an online experiment, which was comparable to effects found in the laboratory. Thus, speech onset latencies can be recorded online with high accuracy.

## Experiment 1

### Method

#### Participants

Ninety-two native English speakers (28 males, 64 females; *M*age = 25.50 years) were recruited from Prolific Academic and participated in exchange for £1.25. All participants resided in the United Kingdom and had a minimum 90% satisfactory completion rate from prior assignments. Participants reported no known speaking, reading, or hearing impairments. The experiment was approved by the University of Edinburgh ethics committee, all experimental methods were performed in line with the University of Edinburgh’s ethical guidelines. and all participants provided informed consent.

#### Materials

We selected 46 questions (see Appendix A) using an online pre-test administered using Google Forms, in which 20 participants (7 males, 13 females; *M*age = 18.60 years) read 98 questions and were instructed to: “Answer each question to the best of your ability. This answer can be a single word or it can be longer and contain any number of words”. Participants provided typed responses. There were two versions of each question: one which we judged to contain the critical content early (i.e., several words before sentence end), and one late (last word). Participants were randomly assigned to one of two stimulus lists that contained one version of each question (10 participants per list) and equal numbers of early and late questions.

Using the pre-test responses, we calculated descriptives for the answers. We analysed the data using ANOVAs and calculated Bayes Factors (BF) for each independent variable using the BayesFactor package (version 0.9.12-4.7). We compared an intercept only model (M0) to a model including the variable of interest (e.g., Critical Content; M1). A Bayes Factor of approximately 1 indicates no evidence in favour of either model. As the Bayes Factor increases over 3, evidence in favour of M1 strengthens; as the Bayes Factor increases under 0.33, evidence strengthens in favour of M0 (e.g.,^31,32^).

We calculated the average word length of answers provided for each of the 46 selected questions. Answers were longer in the long-answer than the short-answer condition (*p* < 0.001; BF > 100; see Table 2). Importantly, there was no difference in the average answer length for questions in the early and late conditions (*p* = 0.78; BF = 0.22) and no interaction between Answer Length and Critical Content (both *p*s = 0.75; BF = 0.22). The standard deviation (SD) of answer word length was low in all conditions, indicating that answers in a particular condition tended to be of a similar word length.

**Table 2:** Means (and SDs) of answer word length, question LSA, answer LSA, and question duration (ms) for stimuli in the four conditions in Experiment 1.

Answer length	Critical content	Answer word length	Question LSA^a^	Answer LSA^b^	Question duration
Short	Early	1.01 (0.03)	0.96 (0.08)	0.97 (0.04)	4145 (1348)
	Late	1.01 (0.03)	0.92 (0.09)	0.96 (0.06)	4109 (1387
Long	Early	3.67 (0.73)	0.91 (0.10)	0.93 (0.08)	4469 (1522)
	Late	3.61 (0.74)	0.91 (0.11)	0.95 (0.07)	4416 (1586)

^a^Average LSA value over comparisons between all answers provided to a particular question. Values closer to 1 indicate that participants tended to provide similar answers.

^b^Average LSA value over comparisons between the most frequent answer and all others. Values closer to 1 indicate that participants tended to provide similar answers.

We assessed the predictability of answers in the four conditions to ensure that participants would produce similar answers to the questions in the main experiment, and so any differences in answer times could not be attributed to answers being unpredictable. Assessing predictability also allowed us to ensure that participants would know the answers to the questions. We used Latent Semantic Analysis (LSA;^33^) matrix comparisons with the general reading corpus provided by Colorado University Boulder (http://lsa.colorado.edu). LSA determines the similarity of words and phrases by calculating the extent to which they occur in the same contexts. Values range from 1 (answers occur in identical contexts) to − 1 (answers occur in completely different contexts). Using these comparisons, we calculated two values. First, we determined the predictability of each question by averaging over the LSA values for all pairwise comparisons between answers, which allowed us to assess the extent to which a question predicted a particular answer. Average question LSA did not differ for the early and late conditions (*p* = 0.45; BF = 0.28) and there was no interaction between Answer Length and Critical Content (*p* = 0.53; BF = 0.26). The frequentist test showed no evidence that the short and long conditions differed in question LSA (*p* = 0.14) and the Bayesian analysis showed no evidence for the alternative or the null (BF = 0.58), Thus, we conclude that there was no consistent evidence that the conditions differed in question LSA.

Next, we determined the similarity of each answer by averaging over the LSA scores for all comparisons between one answer and every other answer to the same question, which allowed us to assess the extent to which participants’ answers overlapped. Average answer LSA did not differ for the early and late conditions (*p* = 0.82; BF = 0.22). The frequentist test showed no evidence that the short and long conditions differed in answer LSA (*p* = 0.06), nor that there was an interaction between Answer Length and Critical Content (*p* = 0.31) and the Bayesian analysis showed no evidence for the alternative or the null in both cases (short/long BF = 0.35; interaction BF = 1.14). Thus, we again concluded that there was no consistent evidence that the conditions differed in average answer LSA. Importantly, LSA values were high (see Table 3). The high question LSA values suggest that our questions were predictable, and tended to elicit similar answers from participants. The high answer LSA values confirm this conclusion, and suggest that participants tended to produce similar answers to the questions. Thus, we assumed the questions were easy to answer.

**Table 3:** Means (and SDs) of answer word length, question LSA, answer LSA, and question duration (ms) for stimuli in the four conditions in Experiment 2.

Answer length	Critical content	Answer word length	Question LSA^a^	Answer LSA^b^	Question duration
Short	Early	1.01 (0.04)	0.94 (0.10)	0.96 (0.07)	4308 (795)
	Late	1.02 (0.05)	0.93 (0.11)	0.95 (0.08)	4212 (845)
Long	Early	3.10 (0.94)	0.90 (0.14)	0.93 (0.09)	4806 (1147)
	Late	3.06 (0.88)	0.88 (0.14)	0.93 (0.09)	4760 (1102)

^a^Average LSA value over comparisons between all answers provided to a particular question. Values closer to 1 indicate that participants tended to provide similar answers.

^b^Average LSA value over comparisons between the most frequent answer and all others. Values closer to 1 indicate that participants tended to provide similar answers.

Questions were recorded by a native English female speaker. Recordings were between 1635 and 7077 ms (see Table 2). The early and late conditions did not differ in duration (*p* = 0.89; BF = 0.22) and there was no interaction between Critical Content and Answer Length (*p* = 0.98; BF = 0.22). The frequentist test showed no evidence that the short and long conditions differed in duration (*p* = 0.31) and the Bayesian analysis showed no evidence for the alternative or the null (BF = 0.35). Thus, there was no consistent evidence that the conditions differed in duration.

#### Design

Answer Length (short vs. long) was manipulated within participants, but between items. Critical Content (early vs. late) was manipulated within participants and items, and so there were two versions of each question. Participants were assigned to one of two stimulus lists (each containing 46 questions), so that they heard one version of each question, but a similar number of early and late questions which required short and long answers.

#### Procedure

We administered the experiment online. Stimulus presentation and data recording were controlled by jsPsych (version 6.0.5;^34^). Participants were told that they would be listening to audio stimuli and would have their voice recorded, so they were encouraged to complete the experiment in a quiet environment using their computer speakers. Before beginning the experiment, participants checked their microphone was clearly recording their answers. They read the sentence “This experiment is fun” and then listened to their audio recording to ensure they could hear themselves clearly.

Participants pressed the spacebar to begin question playback. A fixation cross (+) appeared 500 ms before question onset, and the fixation cross turned red as audio playback began. Participants were instructed to: “Answer the question with the word or words that you think are most appropriate as quickly as possible. Do not wait until the speaker has finished the question and has stopped speaking. Instead, you should answer as soon as you expect the speaker to finish the question”. Thus, participants were encouraged to prepare a response as soon as possible (rather than simply wait for the end of the question). Participants spoke into their microphone and pressed the space bar after answering the question. Audio recording began as soon as question playback started, and ended once participants pressed the space bar. Participants completed four initial practice trials to familiarise themselves with the experimental procedure before they were presented with the 46 questions.

### Data analysis

Answer times were calculated manually in Praat and were the interval between question end (calculated by determining the question’s duration) and the beginning of the answer, ignoring any non-speech sounds such as audible in-breaths but including disfluencies (e.g., *uhh*). We discarded: (1) seven (0.17%) answer times greater than 10,000 ms, as they were clear outliers and unlikely to reflect normal language processing; (2) 145 (3.43%) answers because we could not determine what the speaker said; and (3) 155 (3.66%) answers because participants produced a disfluency or a non-speech sound before producing the answer. Additionally, we coded the accuracy of participants’ answers. Answers were considered accurate if they matched the answer that most participants produced in the pre-test (e.g., *Twenty fifth of December* in response to the question *Which date, when Santa Claus visits, is also a national holiday?*), but we also accepted variations in wording (e.g., *December twenty fifth*). We removed any inaccurate responses from further analyses, since they represented a small subset of the data (585 answers, 96 early-short, 167 early-long, 125 late-short, 197 late-long; 13.82%). Note, however, that we conducted a comparable LMM analysis on all trials (accurate and inaccurate) and found the same pattern of results.

We evaluated the effects of Critical Content and Answer Length on answer times using linear mixed effects models with the *lmer* function of the *lme4* package (version 1.1-31) in RStudio (version 2022.12.0+353). To check assumptions of normality, we visually inspected QQ-plots. The distribution of residuals was acceptable, with the majority following a normal distribution. The plot did show a worse fit for the longest answer times, likely because we selected a long cut-off value for discarding outliers (10,000 ms). However, log-transforming the data led to worse fit and so we modelled the raw answer times.

Answer times were predicted by Critical Content (reference level: late vs. early), Answer Length (reference level: long vs. short), and their interaction (see Table A2 in the Appendix for the full model formula and output). These predictors were contrast coded (− 0.5, 0.5) and centered. Previous research suggests answer times are affected by stimulus duration (e.g.,^4^), and so we also included (centered) Question Duration as a fixed effect in our analysis. A model using the maximal random effects structure returned a singular fit error, likely because the interaction between Critical Content and Answer Length explained very little variance. As a result, our random effects structure included by-participant random effects for Answer Length and Critical Content, and by-item random effects for Critical Content. Correlations among random effects were fixed to zero to aid model convergence^35^.

To preview our results, we found no evidence for an interaction between Critical Content and Answer Length. We calculated Bayes Factors for all predictors by fitting Bayesian mixed effects models using the *brms* package (version 2.18.0). We calculated Bayes Factors by comparing a model with the predictor of interest (e.g., Critical Content; M1) to a reduced model without this predictor (M0).

We fitted models using informative priors, based on our expectation from previous studies. All priors were set using a normal distribution. Based on our pilot study, we expected response times to average around 600 ms, with some variability, and so for the Intercept we set a prior with a mean of 600 ms and a standard deviation of 200 ms. We expected a negative effect of Critical Content (i.e., faster responses for short than long answers) and we expected this effect to be around 400 ms based on our pilot experiment. Thus, we set a prior with a mean of − 400 ms and a standard deviation of 200 ms for the effect of Critical Content.

We also expected a negative effect of Answer Length (i.e., faster responses for short than long answers). In our pilot experiment, the difference between the short and the long-answer conditions was 53 ms, and this effect did not reach significance. We expected a significant Answer Length effect in this study because we strengthened our length manipulation, but we expected it to be smaller than the effect of Critical Content. In a previous study using a similar manipulation, we found an Answer Length of 264 ms^36^. Thus, we set a prior with a mean of − 250 ms and a standard deviation of 200 ms for Answer Length. We did not expect an interaction between these two predictors, and so we set a prior with a mean of 0 ms and a standard deviation of 200 ms for the interaction coefficient. For the standard deviation parameter, we set a prior with a mean of 0 ms and a standard deviation of 50 ms; for sigma, we set a prior with a mean of 0 ms and a standard deviation of 100 ms. We did not calculate a Bayes Factor for Question Duration because we included this predictor as a control variable; we were not interested in whether it affected response times.

We first fitted a model that simulated data from the actual priors, and then visualised the distribution of effects to ensure they matched our expectations. Once we confirmed that the priors seemed plausible, we fitted models with the actual data. Bayes Factors are sensitive to the choice of prior, and so we also conducted a sensitivity analysis^37^. We kept the same means as defined in our informative priors, but we changed the standard deviation of each parameter. In particular, we defined a range of priors with standard deviations from 300 to 1000 ms in increments of 100 ms representing increasingly looser priors and increasing uncertainty about the effect.

For each predictor, we report coefficient estimates (*b*), standard errors (*SE*), and t-values for each predictor. We assume that a *t*-value of 1.96 or greater indicates significance at the 0.05 alpha level^38^. For Bayesian analysis, we report Bayes Factors (BF) from the informative model only, but we report whether the BF was consistent across the sensitivity analysis. The raw data and analysis scripts are available at: https://osf.io/72ahq/.

## Results

**Figure 1:**
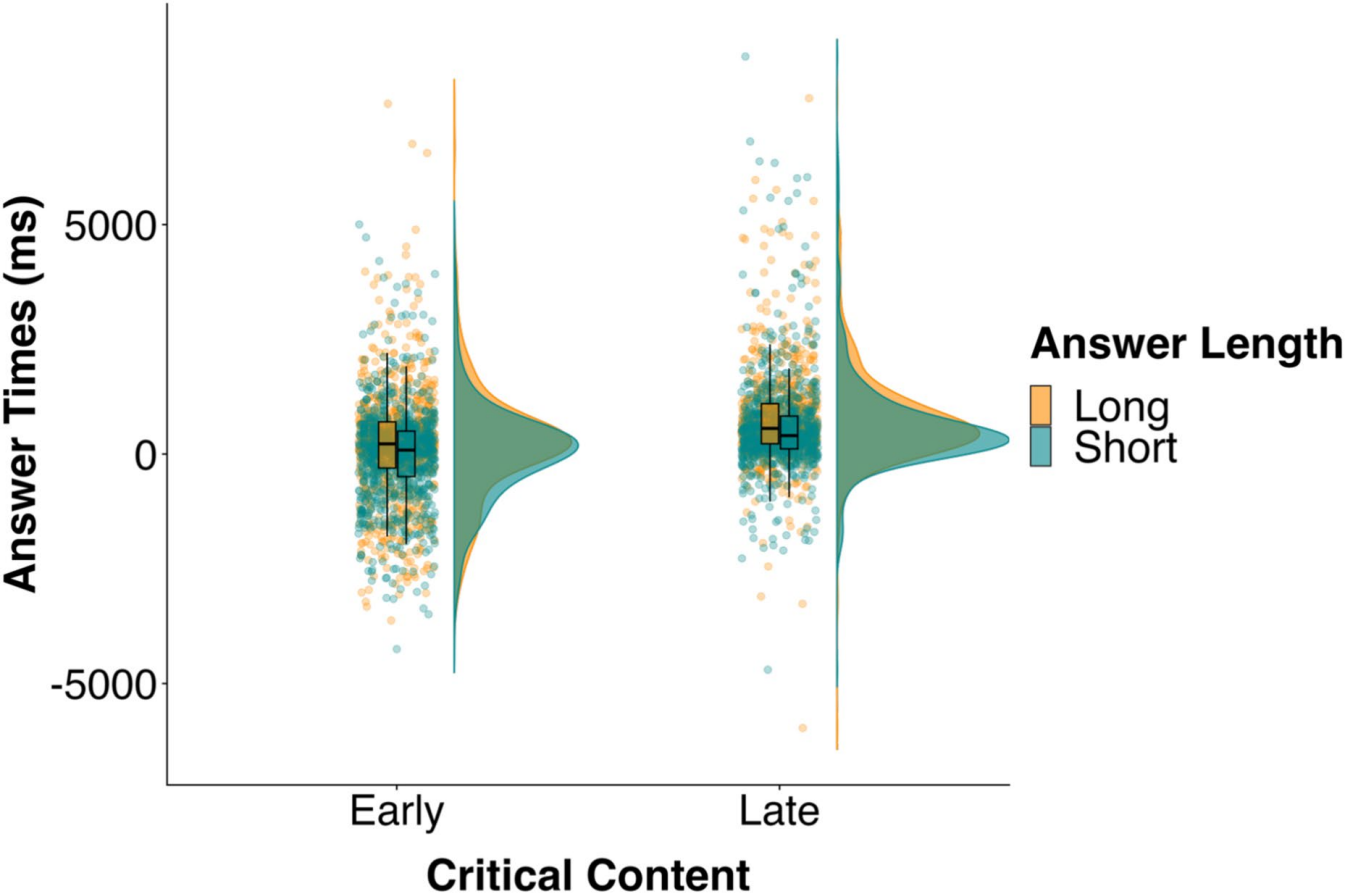
Distribution and mean of answer times (ms) in the four conditions in Experiment 1. Individual dots show individual data points.

On average, participants answered 367 ms after question end, and 88% of the answers occurred within 2000 ms of question end (Fig. 1). Participants responded before the question end on 933 of the trials (28%), with 396 (42%) in the early short condition, 291 (31%) in the early long condition, 151 (16%) in the late short condition, and 95 (10%) in the late long condition.

Participants answered more quickly when the Critical Content occurred early (*M* = 108 ms) rather than late (*M* = 661 ms; *b* = − 608.31, *SE* = 76.90, *t* = − 7.91, BF > 100). The BF showed consistent evidence for the alternative hypothesis over the null in the sensitivity analysis (all BFs > 100). Participants also answered more quickly when the answer was short (*M* = 270 ms) rather than long (*M* = 491 ms; *b* = − 234.13, *SE* = 100.48, *t* = − 2.33, BF = 1.67). Note that the BF showed no evidence for either the null or the alternative hypothesis, and this was the case across the sensitivity analysis (BFs between 0.37 and 1.27). Importantly, there was no interaction between Critical Content and Answer Length (*b* = 40.13, *SE* = 142.37, *t* = 0.28, BF = 0.17) and the BF showed consistent evidence for the null over the alternative hypothesis in the sensitivity analysis (all BFs < 0.10). Finally, longer questions elicited earlier answers than shorter questions (*b* = − 205.01, *SE* = 142.37, *t* = 0.28).

## Discussion

In Experiment 1, we investigated whether speakers prepared both the content and the form of their answers early (early-form account), or just the content (late-form account). Participants answered more quickly when the critical content necessary for answer preparation occurred early (e.g., *Which animal ****barks**** and is also a common household pet*?) rather than late (e.g., *Which animal is a common household pet and also ****barks****?*), and the Bayes Factors showed consistent support for this effect. Participants also answered more quickly when to-be-prepared answers were short rather than long. However, this effect should be interpreted with caution because the Bayes Factors did not show support for either the null or the alternative hypothesis. Importantly, there was no interaction between these two factors and the Bayes Factor strongly supported this null effect.

These results do not provide clear support for either the early- or the late-form account. In particular, the late-form account predicts no interaction between Critical Content and Answer Length, but it does predict that there should be two main effects. The early-form account, in contrast, predicts two main effects and an interaction. Thus, we have strong evidence that participants prepared the content of their answer early, but we cannot make any strong claims about whether they prepared (at least) some of the form of their responses.

One possible explanation for our unclear results is because our strong effect of Critical Content could have occurred because questions in the early condition were easier to answer than those in the late condition. In particular, 28 of the questions had slightly different wording in the early versus the late conditions (e.g., *Which city is the capital of ****Scotland**** and is also home to a castle*? vs. *Which city, that is also home to a castle, is the capital of ****Scotland****?*), which may have made some early questions easier to understand and answer than some of the late questions. Note that an equal number of short-answer and long-answer questions differed in their wording (14 each).

This explanation seems unlikely, given that the four conditions did not differ in question or answer LSA and this measure provides an index of how easy the question is to answer: if a question has lower LSA, then it would suggest that different participants answer it in many different ways, leading to difficulty determining the answer. Nevertheless, we conducted Experiment 2 to rule out the possibility that the Critical Content effect and the lack of Answer Length effect can be attributed to differences in understanding. In particular, we used identical wording in questions in the early (e.g., *Which city is the capital of ****Scotland**** and is home to a castle?)* and late conditions (e.g., *Which city is home to a castle and is the capital of ****Scotland***?).

## Experiment 2

### Method

#### Participants

Experiment 2 was pre-registered (https://osf.io/rdnmj). Following this pre-registration, we selected 62 participants (48 females, 12 males, 2 NA; *M*age = 28.27 years) for analysis from a sample of 79 native English speakers who were recruited using the same terms as Experiment 1. We discarded data from 17 participants, either because their audio responses were not clearly audible or because they listened to the questions using headphones, which made it impossible to determine answer times. We recruited fewer participants for Experiment 2 because we increased the number of items in the study (from 46 to 70), and thus we still had a similar number of datapoints (4340 in Experiment 2; 4232 in Experiment 1) despite recruiting fewer participants.

#### Materials, design, and procedure

We selected 70 questions using the same procedure as Experiment 1 with 20 new participants (16 females, 4 males; *M*age = 26.45 years) who were presented with 82 questions. Some of these questions were new, while others identical to those used in Experiment 1 or were taken from Experiment 1 and re-worded (see Appendix).

We calculated stimuli descriptives using the same procedure as Experiment 1. Answers were significantly longer in the long-answer than the short-answer condition (*p* < 0.001; BF > 100; see Table 3), but there was no difference in the average length of answers for questions in the early and late conditions and no interaction between Answer Length and Critical Content (all *p*s > 0.84; both BFs = 0.18). Average question (*p* = 0.79; BF = 0.19) and answer (*p* = 0.70; BF = 0.20) LSA did not differ for the early and late conditions and there was no interaction between Critical Content and Answer Length (question LSA *p* = 0.96; BF = 0.18; answer LSA *p* = 0.95; BF = 0.19). The frequentist test showed no difference in question and answer LSA for the short and long conditions (both *p*s = 0.06) and the Bayesian analysis showed no evidence for either the null or alternative hypothesis (question LSA BF = 1.00; answer LSA BF = 0.98). Thus, we concluded that there was no consistent evidence that the conditions differed in average question or answer LSA. Importantly, these values were high (Table 3), suggesting questions were easy to answer. Questions were recorded using the same procedure as Experiment 1. Recordings were between 2933 and 7661 ms (Table 3). Questions in the short-answer condition were shorter than in the long-answer condition (*p* < 0.001; BF = 15.84), but the other conditions did not differ in duration (*p*s > 0.67; BFs between 0.18 and 0.20). Research has shown that participants are faster to answer questions that are longer in duration (e.g.,^4^), and so this duration effect may mean that participants are faster to produce long-answer than short-answers. Importantly, this pattern runs counter to what we would expect if participants are affected by answer length. The design and procedure for the experiment were identical to Experiment 1.

### Data analysis

We analysed the data using the same procedure as Experiment 1. We discarded: (1) 82 (1.89%) answers because we could not determine what the speaker said; (2) 375 (8.64%) answers because the participant produced a disfluency or a non-speech sound before producing the answer; and (3) 416 (85 early-short, 121 early-long, 82 late-short, 128 late-long; 9.59%) answers because they were inaccurate. Note that in our pre-registration, we planned to analyse the number of disfluencies participants produced and their response accuracy before analysing response times for the fluent and accurate trials. We did not carry out this analysis, however, because they represented such a small subset of the data and they were not critical for testing the early- and late-form accounts. Note, however, that we conducted a comparable LMM analysis on all trials (accurate and inaccurate). Here, there was no longer an interaction between Critical Content and Answer Length (*b* = 204.45, *SE* = 121.67, *t* = 1.68), perhaps because participants were more inaccurate (and thus slower) overall in the long conditions.

We fitted models using the same procedure as Experiment 1. A model using the maximal random effects structure returned a singular fit error, likely because the interaction between Critical Content and Answer Length explained very little variance. As a result, our random effects structure included by-participant random effects for Answer Length and Critical Content, and by-item random effects for Critical Content. Correlations among random effects were fixed to zero to aid model convergence^35^. For the Bayesian analysis, we used the same procedure as Experiment 1, but we used the estimates from Experiment 1. In particular, we set a prior with a mean of 350 ms and a standard deviation of 200 ms for the Intercept. For the Critical Content effect, we set a prior with a mean of − 550 ms and a standard deviation of 200 ms. For the Answer Length effect, we set a prior with a mean of − 250 ms and a standard deviation of 200 ms. All other priors were the same as Experiment 1. Note that we did not plan to fit these Bayesian models in our preregistration, but we included them in our analyses to add further weight to our null effects.

### Results

**Figure 2:**
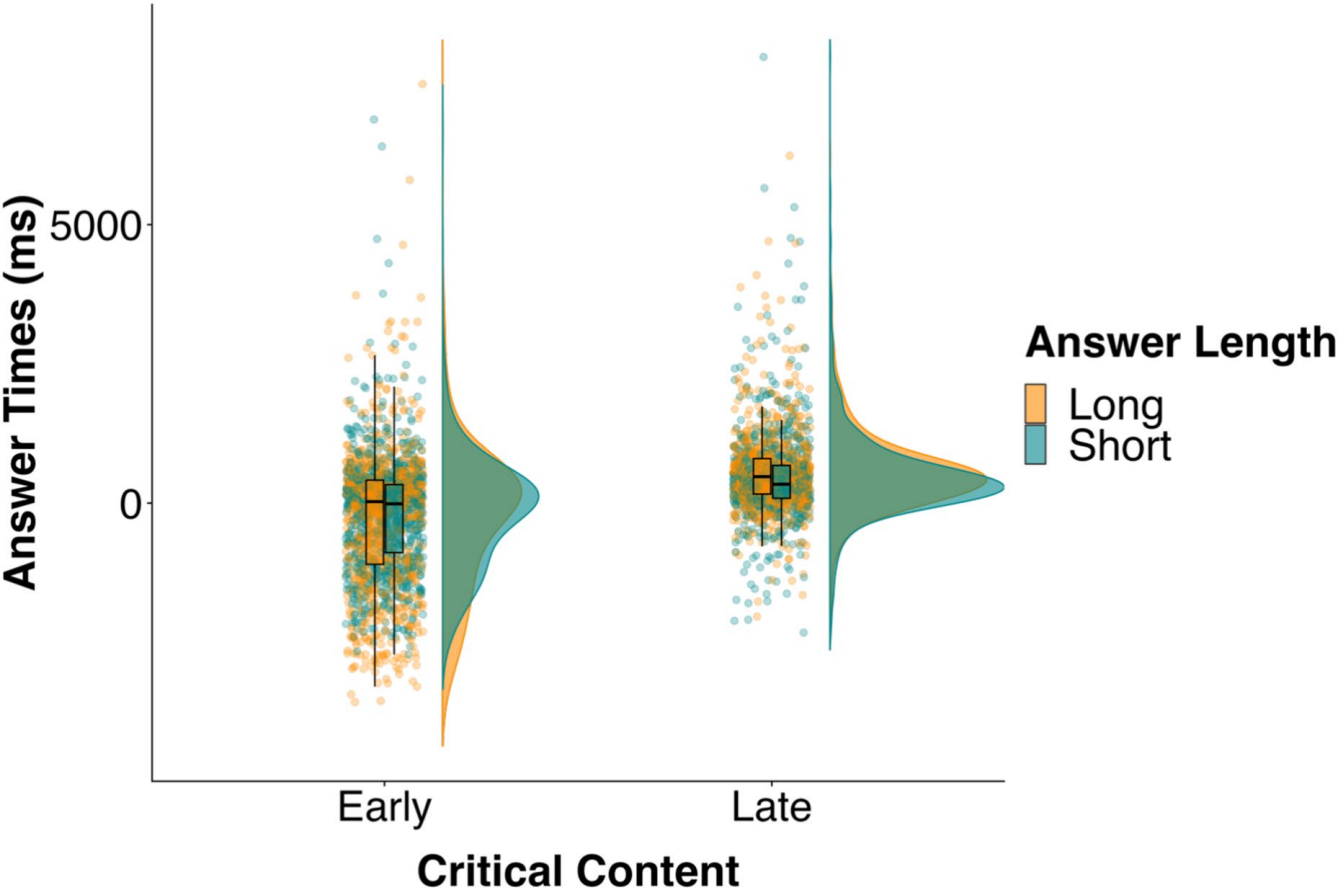
Distribution and mean of answer times (ms) in the four conditions in Experiment 2. Individual dots show individual data points.

On average, participants answered 111 ms after question end, and 93% of the answers occurred within 2000 ms of question end (Fig. 2). Participants responded before the question end on 1191 of the trials (34%), with 497 (42%) in the early short condition, 423 (36%) in the early long condition, 154 (13%) in the late short condition, and 117 (10%) in the late long condition.

Participants answered more quickly when the Critical Content occurred early (*M* = − 216 ms) rather than late (*M* = 505 ms; *b* = − 735.68, *SE* = 80.00, *t* = − 9.20, BF > 100), consistent with Experiment 1. The BF showed consistent evidence for the alternative hypothesis over the null in the sensitivity analysis (all BFs > 100). Note, however, that the answer times in this experiment contain many more negative responses than Experiment 1, especially in the early conditions, suggesting participants tended to produce an answer before the question ended. Inconsistent with Experiment 1, there was no difference in answer times for the short-answer (*M* = 121 ms) and long-answer conditions (*M* = 112 ms; *b* =− 100.34, *SE* = 79.16, *t* = − 1.27, BF = 0.09), and the BF consistently supported the null hypothesis over the alternative in the sensitivity analysis (all BFs < 0.08). Note that the beta coefficient is much larger than the mean difference between the two conditions because this coefficient is adjusted for effects of Question Duration. Participants were faster to answer questions that were longer in duration (*b* = − 194.43, *SE* = 39.22, *t* = − 4.96).

We found a marginally significant interaction between Critical Content and Answer Length (*b* = 236.09, *SE* = 117.93, *t* = 2.00, BF = 1.18). However, the BF did not provide consistent evidence for either the alternative or the null hypothesis, and even supported the null at priors with SDs larger than 700 ms (all BFs between 800 and 1000 ms were less than 0.27; BFs between 300 and 700 ms were between 0.68 and 0.33). Nevertheless, we followed up this interaction by fitting separate models to the early and late Critical Content conditions, testing for an effect of Answer Length. In these models, we included Answer Length and Question Duration as fixed effects to control for any differences in question duration. Note that we do not report these effects in the “Results” section because they were not critical for our analysis. We also do not report BFs for these analyses because the interaction was marginal, and so the post-hoc tests were exploratory. We included only by-participant and by-item intercepts because including by-participant random effects for Answer Length resulted in a singular fit error. We found no effect of Answer Length in the early (*b* = − 44.67, *SE* = 96.20, *t* = − 0.46) or late (*b* = − 143.29, *SE* = 94.60, *t* = − 1.51) Critical Content conditions, but the Answer Length effect was larger (a coefficient of − 143.29) in the late than in the early Critical Content condition (a coefficient of − 44.67).

### Discussion

In Experiment 2, we further investigated whether speakers prepared both the content and the form of their answers early (early-form account), or just the content (late-form account). As in Experiment 1, participants answered more quickly when the critical content necessary for answer preparation occurred early rather than late. Unlike Experiment 1, however, there was no difference in answer times when to-be-prepared answers were short compared to when they were long, and the Bayes Factors showed consistent support for this null effect. We found a marginally significant interaction between these two factors, such that the answer length effect was larger in the late than the early condition. This pattern of results is consistent with the early-form account. However, the Bayes Factors did not provide consistent support for this effect, and sometimes even supported the null, suggesting that this interaction should be interpreted with caution.

**Figure 3:**
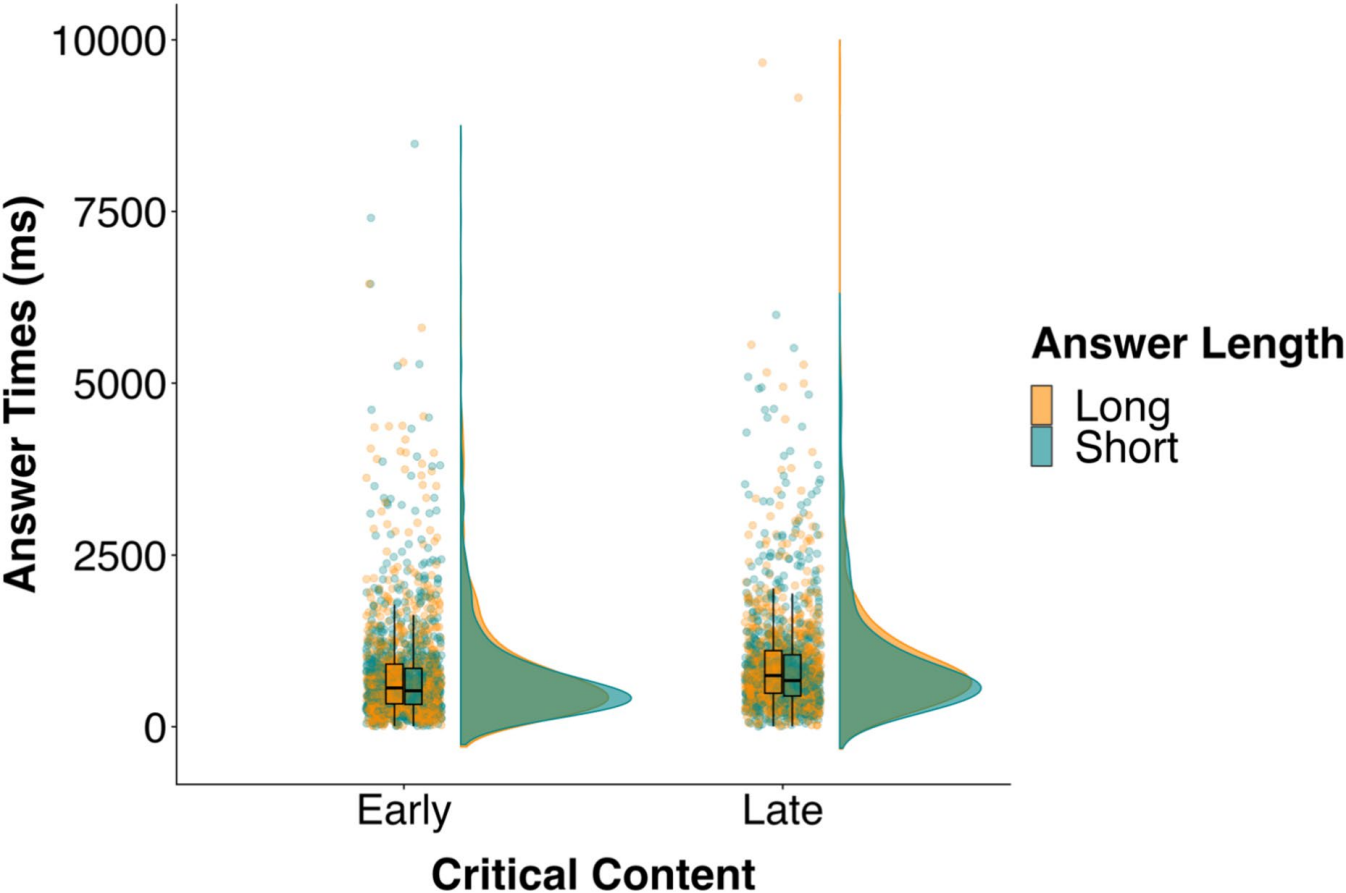
Distribution and mean of answer times (ms) in the four conditions in Experiment 3. Individual dots show individual data points.

How can we interpret these results? Our findings could be mixed because participants in this experiment tended to respond before the question end, which was not the case in Experiment 1. In fact, Fig. 3 shows that average answer times in the early condition were negative. As a result, the participant tended to answer before the speaker had finished the end of their question. Such negative responses complicate the interpretation of our results—our hypotheses concern the *speed* with which participants respond, but it is not clear that a *faster* response is necessarily better when response times are negative. In these cases, participants respond faster, but they also overlap with the previous speaker and overlaps can disrupt the flow of interaction. Furthermore, such overlaps make it difficult to interpret our results. For example, we would expect participants to respond more quickly to early-short than early-long questions under the late-form account, and this would suggest participants experienced more difficulty planning a long than a short answer. We do not find this pattern of results—Fig. 3 shows that participants were faster for early-long than early-short questions. However, participants do respond closer to the question end in the early-short than the early-long condition and responding with less overlap (but slower) may be better. Thus, it is unclear what these negative responses actually mean. As a result, we conducted Experiment 3 to further test the early- and late-form accounts. In particular, we used the same materials and procedure as Experiment 2, but we instructed participants to answer only once the speaker had reached the end of the question, and so we could minimise the occurrence of negative responses.

## Experiment 3

### Method

#### Participants

Experiment 3 was pre-registered (https://osf.io/9qs6w). Following this pre-registration, we selected 62 participants (52 females, 10 males; *M*age = 28.79 years) for analysis from a sample of 112 native English speakers who were recruited using the same terms as Experiment 1. Note that we analysed the data from only 62 participants because an update to most internet browsers meant that most browsers now implement noise cancellation during audio recording. As a result, many participants were unable to record the audio for a full trial, meaning that audio was available for either the question or the answer, but often not both. The 62 participants we analysed were using older internet browsers and could record both the question and the answer, and so we could determine answer times.

#### Materials, design, and procedure

The materials, design, and procedure were identical to Experiment 2, with the exception that participants were instructed to wait until the speaker had reached the end of their question before providing an answer.

### Data analysis

We analysed the data using the same procedure as Experiment 2. We discarded: (1) 42 (0.97%) answers because we could not determine what the speaker said; (2) 293 (6.75%) answers because the participant produced a disfluency or a non-speech sound before producing the answer; (3) 430 (89 early-short, 120 early-long, 89 late-short, 132 late-long; 9.91%) answers because they were inaccurate; (4) five (0.12%) answers because they were longer than 10,000 ms; and (5) 79 (1.82%) answers because participants answered before the question end. We fitted models using the same procedure as Experiment 2. Note that even though we pre-registered this analysis, we again did not analyse the number of disfluencies participants produced or the accuracy of their responses because it accounted for such a small proportion of the data. Note, however, that we conducted a comparable LMM analysis on all trials (accurate and inaccurate) and found the same pattern of results. We expected participants in this experiment to be slower to respond than those in Experiment 2. Thus, we calculated Bayes Factors using the same priors as Experiment 1.

### Results and discussion

On average, participants answered 830 ms after question end, and 94% of the answers occurred within 2000 ms of question end (Fig. 3). Note that answer times in this experiment are much longer than those in Experiments 1 and 2, likely because we explicitly asked participants to answer only once the speaker reached the end of the question. Thus, there were fewer negative responses in this experiment (79; 1.82%), compared to 435 (10.28%) in Experiment 1 and 1271 (29.29%) in Experiment 2.

As in Experiments 1 and 2, participants answered more quickly when the Critical Content occurred early (*M* = 747 ms) rather than late (*M* = 920 ms; *b* = − 180.91, *SE* = 31.99, *t* = − 5.66, BF = 81.08) and the BF showed consistent evidence for the alternative hypothesis over the null in the sensitivity analysis (all BFs > 10). The Critical Content effect was much smaller in this experiment (*M*difference = 173 ms) than in Experiments 1 (*M*difference = 721 ms) and 2 (*M*difference = 553 ms), likely because participants were able to produce their answers before the question end in Experiments 1 and 2. Importantly, the Critical Content effect in this experiment suggests that there is a response time advantage to encountering the critical content early, even when speakers have to wait until question end to produce their response.

There was no difference in answer times for the short-answer (*M* = 808 ms) and long-answer conditions (*M* = 846 ms; *b* = − 29.27, *SE* = 59.05, *t* = − 0.50, BF = 0.08), consistent with Experiment 2, and the BF showed consistent evidence for the null (all BFs < 0.07). Importantly, and as in Experiment 1, there was no interaction between Critical Content and Answer Length (*b* = − 8.17, *SE* = 60.93, *t* = − 0.13, BF = 0.07) and the BF consistently supported the null (all BFs < 0.04). There was also no effect of Question Duration (*b* = 3.10, *SE* = 27.58, *t* = 0.11).

Thus, we found that participants in Experiment 3 answered questions more quickly when the Critical Content necessary for response preparation was available early rather than late, suggesting speakers prepared the content of their answers early. We found no evidence that answer times were affected by the length of the to-be-prepared answer, and there was no interaction between Critical Content and Answer Length.

## Combined analysis

In three experiments, we investigated whether participants prepared the content of their answers early (late-form account), or whether they prepare both the content and the form early (early-form account). The late-form account predicts that participants should be faster to answer questions in which the Critical Content is available early, rather than late. It also predicts that participants should be faster to answer questions when the to-be-prepared answer is short (one word) rather than long (multiple words). The early-form account makes the same predictions, but it also predicts an interaction between Critical Content and Answer Length: there should be a larger effect of Answer Length when the Critical Content is available late rather than early.

So far, we have not found strong support for either of these accounts. In particular, we have found a strong effect of Critical Content in all three experiments, but no strong effect of Answer Length or an interaction. It is possible that our mixed results can be attributed to a lack of power—for example, we may not have had sufficient power to detect a significant effect of Answer Length and so it was unreliable in Experiment 1 and non-significant in Experiments 2 and 3. This explanation seems unlikely, given that we have found a significant effect of Answer Length in previous studies using a similar manipulation with fewer participants^36^. To address this issue, however, we conducted a combined analysis of Experiments 1 and 2. We did not include Experiment 3 in this analysis because Experiment 3 explicitly asked participants not to respond before the end of the question (and any such responses were excluded from the analysis), while participants in Experiments 1 and 2 could respond before the question end.

We analysed answer times. Although not critical for our predictions, we conducted a comparable analysis on the number of disfluencies participants produced. Participants were disfluent on 409 trials compared to 6805 fluent trials, and they were more disfluent when the Critical Content was available late (*M* = 8%) rather than early (*M* = 4%) and when Answer Length was long (*M* = 8%) rather than short (*M* = 4%). Note that these results should be interpreted with caution, given that there were so few disfluent trials.) and fitted a model using the same procedure as the individual experiments. Again, we did not include by-participant random effects for the interaction between Critical Content and Answer Length because the model returned a singular fit error. Thus, the model included by-participant random effects for Critical Content and Answer Length, and by-item random effects for Critical Content. Although some of the questions in the early and late conditions differed in their wording in Experiment 1, this difference did not explain the Critical Content effect in Experiment 2 (i.e., it was still present even when the questions were identical in their wording).

We had also planned to investigate the interaction between Experiment, Critical Content, and Answer Length. However, testing for this interaction rests on finding an interaction in the individual experiments. We did find this interaction in the LMM analysis for Experiment 2, but it was marginally significant and the Bayes Factors did not support this effect. Thus, we simply tested for effects of Critical Content, Answer Length, and their interaction, pooling the data together to increase our power for detecting such effects. We calculated Bayes Factors using the same procedure as our individual analyses, but we used the same priors as Experiment 1.

As in our previous analyses, participants answered more quickly when the Critical Content was available early (*M* = − 82 ms) rather than late (*M* = 588 ms; *b* = − 655.20, *SE* = 58.29, *t* = − 11.24, BF > 100) and the BF showed consistent support for the alternative hypothesis over the null (all BFs > 100). Participants also answered more quickly when the to-be-prepared answer was short (*M* = 382 ms) rather than long (*M* = 484 ms; *b* = − 221.53, *SE* = 48.27, *t* = − 4.59, BF > 100) and the BF again showed consistent support for the alternative hypothesis (all BFs > 100). Participants were also slower to answer questions that were longer in duration (*b* = − 175.40, *SE* = 17.12, *t* = − 10.25). Importantly, there was no interaction between Critical Content and Answer Length (*b* = − 29.01, *SE* = 80.48, *t* = − 0.36, BF = 0.09) and the BF showed consistent support for the null hypothesis (all BFs < 0.07). The findings from the combined analysis are consistent with a late-form account (i.e., effects of Critical Content and Answer Length but no interaction), and suggest that participants prepared the content of their answers early, but their form late.

## General discussion

In three experiments, we used a question-answering task to investigate what aspects of their response speakers prepare early. We contrasted an early-form account, which claims that speakers prepare both the content and (at least some of) the form of their answer early, with a late-form account, which claims that speakers fully prepare only content early. We manipulated the availability of the critical content (here, *barks*) necessary for answer preparation: it was available either early (e.g., *Which animal barks and is also a common household pet*?) or late (e.g., *Which animal is a common household pet and also barks*?). To determine whether participants prepared the form of their answers early, we manipulated the length of the to-be-prepared answers, so that they were either short (single word) or long (multi-word).

In all three experiments, participants answered more quickly when the critical content was available early rather than late. In the individual experiments, we did not find convincing evidence that participants were slower to produce long-answers than short-answers (this effect was significant only in Experiment 1, but was not supported by the Bayes Factor) and there was no interaction between these two factors. Thus, the findings from the three isolated experiments did not support either the early- or the late-form account. However, a combined analysis of two of the three experiments supported the late-form account. In particular, participants were faster to answer questions when the critical content was available early rather than late and when the answer was short rather than long. Importantly, there was no interaction between the two factors.

Our combined analysis is thus inconsistent with an early-form account, in which speakers prepare the form of their utterances early^13^. Instead, it provides support for a late-form account, in which speakers prepare the content of their answers early but the form late. In particular, this combined analysis is consistent with previous studies investigating the scope of advance planning in monologue, particularly those that have shown that speakers do not prepare the form of their full utterance (e.g.,^21–23^). Our findings suggest that speakers minimise the cognitive demands of overlapping production and comprehension by preparing only the content of their answer early. They thus adopt a strategy that enables partial, but not complete, preparation so that they can still allocate resources to comprehension.

Note, however, that these findings are not clear-cut evidence for the late-form account because we failed to find strong effects of Answer Length in the individual experiments. It is possible that this effect was relatively small. Consistent with this suggestion, our combined analysis showed that the Answer Length effect was less than half the size (a beta coefficient of − 213.36) of the Critical Content effect (a beta coefficient of − 504.68). Thus, we may not have had sufficient power to detect this effect in our individual experiments, and additional research is needed to further test between early- and late-form accounts, determining what aspects of their response speakers plan before speaking. Interestingly, the Answer Length effect observed in our combined analysis is slightly smaller than the length effect we observed in a previous study using a similar manipulation (a beta coefficient of − 277.86) with fewer participants (40) and items (60;^36^). However, participants in our first two experiments could respond before the question end in the early condition, as soon as they heard the critical information necessary for answering (e.g., *barks* in the question *Which animal barks and is also a common household pet?*). Answer times could thus be negative, which may have affected the size of the Answer Length effect. Nevertheless, future research could investigate what these early responses mean, and whether they are necessarily better than later responses.

Our findings provide tentative evidence that speakers prepared the content of their answers early, but not the form. Note that this strategy is unlikely to apply to all types of language production. In some situations, such as in our experiments, preparing the form of the answer in advance may be difficult or the speaker may not have the resources necessary to do so, and so they will prepare the only the content of their utterances early. But in other situations, it is possible that the speaker may allocate more resources to production, planning the form of their response early, together with the content. In fact, research suggests that the extent of preparation is flexible and affects by factors such as time pressure (e.g.,^39^), the familiarity of lexical items (e.g.,^17^), or the ease of constructing a sentence (e.g.,^40^). It is thus possible that speakers will sometimes prepare only the content of their answer early and sometimes prepare both the content and the form.

Our findings are consistent with previous studies demonstrating that speakers prepare the content of their utterances early (e.g.,^4,9^). Such early preparation plays a central role in theories of turn-taking in conversation, which tend to claim that speakers manage the timing demands of conversation by planning an utterance as early as possible (e.g.,^41,42^). It is worth noting, however, that early preparation is unlikely to explain close coordination in all situations. Our questions contained clear cues to the answer, making preparing the content of the utterance relatively easy. Such clear cues are unlikely to be present in natural conversation, which is much less predictable—speakers can often reply in any way they wish. In fact, research has shown that speakers do not always directly respond to each other, as they do in the laboratory^43^. Thus, future research could investigate *which* situations allow for early planning to understand when advance planning supports coordination in conversation.

It is also worth noting that our experiments are not just about producing language, but also about retrieving information from memory. For example, to answer the question *Which animal barks and is also a common household pet*?, the speaker needs to retrieve the concept of a dog from memory before they begin the process of lexicalization and grammatical and phonological encoding. However, memory retrieval is likely to be involved in a wide range of situations in which we produce language; for example, if we are talking to a friend, we may need to recall their preferences or the names of their children. Trials in which participants produced inaccurate answers (including “I don’t know”) are likely to reflect difficulty retrieving the answer from memory. Our analysis excluded these trials, and so our results cannot be solely attributed to difficulty retrieving answers from memory.

In conclusion, our findings demonstrate that participants prepare the content of their answers early, and suggest that they sometimes prepare the form of their answers late. These findings add to the growing body of literature investigating the mechanisms of response planning during turn-taking. They have important implications for understanding how interlocutors manage to coordinate production and comprehension during dialogue.

### Ethical statement

The work reported in this manuscript was approved by the University of Edinburgh ethics committee and all experimental methods were performed in line with the University of Edinburgh ethical guidelines. All participants provided informed consent.

## Supplementary Information

Supplementary Tables.

## Data Availability

Raw data and analysis scripts are available at: https://osf.io/72ahq/. Preregistration for Experiment 2 and 3 can be found at https://osf.io/rdnmj and https://osf.io/9qs6w.

## References

[1] Indefrey, P. & Levelt, W. J. The spatial and temporal signatures of word production components. *Cognition* **92**, 101–144 (2004). 15037128 10.1016/j.cognition.2002.06.001

[2] Stivers, T. *et al.* Universals and cultural variation in turn-taking in conversation. *Proc. Natl. Acad. Sci.* **106**, 10587–10592 (2009). 19553212 10.1073/pnas.0903616106 PMC2705608

[3] Bögels, S. & Levinson, S. C. The brain behind the response: Insights into turn-taking in conversation from neuroimaging. *Res. Lang. Soc. Interact.* **50**, 71–89 (2017).

[4] Corps, R. E., Crossley, A., Gambi, C. & Pickering, M. J. Early preparation during turn-taking: Listeners use content predictions to determine what to say but not when to say it. *Cognition* **175**, 77–95 (2018). 29477750 10.1016/j.cognition.2018.01.015

[5] Lindsay, L., Gambi, C. & Rabagliati, H. Preschoolers optimize the timing of their conversational turns through flexible coordination of language comprehension and production. *Psychol. Sci.* **30**, 504–515 (2019). 30747577 10.1177/0956797618822802

[6] Magyari, L., De Ruiter, J. P. & Levinson, S. C. Temporal preparation for speaking in question-answer sequences. *Front. Psychol.* **2017**, 8. 10.3389/fpsyg.2017.00211 (2017). 10.3389/fpsyg.2017.00211 PMC5318421 28270782

[7] Sjerps, M. J., Decuyper, C. & Meyer, A. S. Initiation of utterance planning in response to pre-recorded and “live” utterances. *Q. J. Exp. Psychol.* **73**, 357–374 (2020). 10.1177/1747021819881265 31544625

[8] Sjerps, M. J. & Meyer, A. S. Variation in dual-task performance reveals late initiation of speech planning in turn-taking. *Cognition* **136**, 304–324 (2015). 25522192 10.1016/j.cognition.2014.10.008

[9] Bögels, S., Magyari, L. & Levinson, L. Neural signatures of response planning occur midway through an incoming question in conversation. *Sci. Rep.* **2015**, 5. 10.1038/srep12881 (2015). 10.1038/srep12881 PMC4525376 26242909

[10] Levelt, W. J. *Speaking: From Intention to Articulation* (MIT Press, 1989).

[11] Raz, N. *et al.* Regional brain changes in aging healthy adults: General trends, individual differences and modifiers. *Cerebral Cortex* **15**, 1676–1689 (2005). 15703252 10.1093/cercor/bhi044

[12] Jongman, S. R., Piai, V. & Meyer, A. S. Planning for language production: The electrophysiological signature of attention to the cue to speak. *Lang. Cogn. Neurosci.* **35**, 915–932 (2020).

[13] Barthel, M. & Levinson, S. C. Next speakers plan word forms in overlap with the incoming turn: Evidence from gaze-contingent switch task performance. *Lang. Cogn. Neurosci.* **35**, 1–20 (2020).

[14] Schnur, T. T., Costa, A. & Caramazza, A. Planning at the phonological level during sentence production. *J. Psycholinguist. Res.* **35**, 189–213 (2006). 16502144 10.1007/s10936-005-9011-6

[15] Schnur, T. T. Phonological planning during sentence production: Beyond the verb. *Front. Psychol.* **2011**, 2. 10.3389/fpsyg.2011.00319 (2011). 10.3389/fpsyg.2011.00319 PMC3208389 22069396

[16] Alario, F. X., Segui, J. & Ferrand, L. Semantic and associative priming in picture naming. *Q. J. Exp. Psychol. Hum. Exp. Psychol.* **53**, 741–764 (2000). 10.1080/713755907 10994228

[17] Konopka, A. E. Planning ahead: How recent experience with structures and words changes the scope of linguistic planning. *J. Mem. Lang.* **66**, 143–162 (2012).

[18] Barthel, M. & Sauppe, S. Speech planning at turn transitions in dialog is associated with increased processing load. *Cogn. Sci.* **2019**, 43. 10.1111/cogs.12768 (2019). 10.1111/cogs.12768 31310021

[19] Fairs, A., Bögels, S. & Meyer, A. S. Dual-tasking with simple linguistic tasks: Evidence for serial processing. *Acta Psychol.* **191**, 131–148 (2018). 10.1016/j.actpsy.2018.09.006 30268022

[20] Bögels, S., Casillas, M. & Levinson, S. C. Planning versus comprehension in turn-taking: Fast responders showed reduced anticipatory processing of the question. *Neuropsychologia* **109**, 295–310 (2018). 29269305 10.1016/j.neuropsychologia.2017.12.028

[21] Ferreira, F. Effects of length and syntactic complexity on initiation times for prepared utterances. *J. Mem. Lang.* **30**, 210–233 (1991).

[22] Meyer, A. S. Lexical access in phrase and sentence production: Results from picture-word interference experiments. *J. Mem. Lang.* **35**, 447–496 (1996).

[23] Smith, M. & Wheeldon, L. Horizontal information flow in spoken sentence production. *J. Exp. Psychol.: Learn. Mem. Cogn.* **30**, 675–686 (2004). 15099135 10.1037/0278-7393.30.3.675

[24] Ferrand, L. *et al.* MEGALEX: A megastudy of visual and auditory word recognition. *Behav. Res. Methods* **50**, 1285–1307 (2017). 10.3758/s13428-017-0943-1 28791657

[25] Hartshorne, J. K., Tenenbaum, J. B. & Pinker, S. A critical period for second language acquisition: Evidence from 2/3 million English speakers. *Cognition* **177**, 263–277 (2018). 29729947 10.1016/j.cognition.2018.04.007 PMC6559801

[26] Anwyl-Irvine, A. L., Dalmaijer, E. S., Hodges, N. & Evershed, J. Online timing accuracy and precision: A comparison of platforms, browsers, and participant’s devices. *PsyArXiv*10.31234/osf.io/jfeca (2020).

[27] Bridges, D., Pitiot, A., MacAskill, M. R. & Peirce, J. W. The timing mega-study: Comparing a range of experiment generators, both lab-based and online. *PeerJ* **2020**, 8. 10.7717/peerj.9414 (2020). 10.7717/peerj.9414 PMC7512138 33005482

[28] Fairs, A. & Strijkers, K. Can we use the internet to study speech production? Yes we can! Evidence contrasting online versus laboratory naming latencies and errors. *Plos One* **2021**, 16. 10.1371/journal.pone.0258908 (2021). 10.1371/journal.pone.0258908 PMC8535377 34679082

[29] Stark, K., van Scherpenberg, C., Obrig, H. & Abdel Rahman, R. Web-based language production experiments: Semantic interference assessment is robust for spoken and typed response modalities. *Behav. Res. Methods* **55**, 236–262 (2023). 35378676 10.3758/s13428-021-01768-2 PMC9918579

[30] Vogt, A., Hauber, R., Kuhlen, A. K. & Abdel Rahman, R. Internet-based language production research with overt articulation: Proof of concept, challenges, and practical advice. *Behav. Res. Methods*10.3758/s13428-021-01686-3 (2021). 34799842 10.3758/s13428-021-01686-3 PMC8604202

[31] Kass, R. E. & Raftery, A. E. Bayes factors. *J. Am. Stat. Assoc.* **90**, 773–795 (1995).

[32] Lee, M. D. & Wagenmakers, E. J. *Bayesian Cognitive Modeling: A Practical Course* (Cambridge University Press, 2014).

[33] Deerwester, S., Dumais, S. T., Furnas, G. W., Landauer, T. K. & Harshman, R. Indexing by latent semantic analysis. *J. Am. Soc. Inf. Sci.* **41**, 391–407 (1990).

[34] De Leeuw, J. R. jsPsych: A javascript library for creating behavioral experiments in a web browser. *Behav. Res. Methods* **47**, 1–12 (2015). 24683129 10.3758/s13428-014-0458-y

[35] Matuschek, H., Kliegel, R., Vasishth, S., Baayen, H. & Bates, D. Balancing Type 1 error and power in linear mixed models. *J. Mem. Lang.* **94**, 305–315 (2017).

[36] Corps, R. E. & Pickering, M. J. Response planning during question-answering: Does deciding what to say involve deciding how to say it?. *Psychon. Bull. Rev.* **31**, 839–848 (2024). 37740119 10.3758/s13423-023-02382-3 PMC11061006

[37] Schad, D. J., Nicenboim, B., Bürkner, P. C., Betancourt, M. & Vasishth, S. Workflow techniques for the robust use of bayes factors. *ArXiv* **2103**, 08744 (2022). 10.1037/met0000472 35266787

[38] Baayen, R. H., Davidson, D. J. & Bates, D. M. Mixed-effects modelling with crossed random effects for subjects and items. *J. Mem. Lang.* **59**, 390–412 (2008).

[39] Ferreira, F. & Swets, B. How incremental is language production? Evidence from the production of utterances requiring the computation of arithmetic sums. *J. Mem. Lang.* **46**, 57–84 (2002).

[40] Wagner, V., Jescheniak, J. D. & Schriefers, H. On the flexibility of grammatical advance planning during sentence production: Effects of cognitive load on multiple lexical access. *J. Exp. Psychol.: Learn. Mem. Cogn.* **36**, 423–440 (2010). 20192540 10.1037/a0018619

[41] Levinson, S. C. & Torreira, F. Timing in turn-taking and its implications for processing models of language. *Front. Psychol.* **2015**, 6. 10.3389/fpsyg.2015.0073 (2015). 10.3389/fpsyg.2015.00731 PMC4464110 26124727

[42] Garrod, S. & Pickering, M. J. The use of content and timing to predict turn transitions. *Front. Psychol.* **2015**, 6. 10.3389/fpsyg.2015.00751 (2015). 10.3389/fpsyg.2015.00751 PMC4463931 26124728

[43] Corps, R. E., Knudsen, B. & Meyer, A. S. Overrated gaps: Inter-speaker gaps provide limited information about the timing of turns in conversation. *Cognition* **2022**, 223. 10.1016/j.cognition.2022.105037 (2022). 10.1016/j.cognition.2022.105037 35123218

