# Supplementary material for: The role of answer content and length when preparing answers to questions
Source: Sci Rep. 2024 Jul 24;14:17110. doi: 10.1038/s41598-024-68253-6 (PMC11269693; doi:10.1038/s41598-024-68253-6)
Supplement: Supplementary file 1 — Supplementary Tables. [file 41598_2024_68253_MOESM1_ESM.docx]

**Appendices**

Table A1. Lists of questions used in all three experiments. Note that the same stimuli were used in Experiments 2 and 3.

| Experiment | Answer Length | Critical Content | Question |
| --- | --- | --- | --- |
| 1 | Short | Early | Which phone brand is represented by a fruit and also makes touchscreen phones? |
|  |  | Late | Which phone brand makes touchscreen phones and also is represented by a fruit? |
|  |  | Early | Which fruit is curved and also yellow? |
|  |  | Late | Which fruit is yellow and also curved? |
|  |  | Early | Which animal meows and also has four legs? |
|  |  | Late | Which animal has four legs and also meows? |
|  |  | Early | Which animal barks and is also a common household pet? |
|  |  | Late | Which animal is a common household pet and also barks? |
|  |  | Early | Which city is the capital of Scotland and is also home to a castle? |
|  |  | Late | Which city, that is also home to a castle, is the capital of Scotland? |
|  |  | Early | Which country, that is home to the pyramids, is located in Africa? |
|  |  | Late | Which country located in Africa is home to the pyramids? |
|  |  | Early | Which city is the capital of the UK and also begins with the letter 'L'? |
|  |  | Late | Which city begins with the letter 'L' and is also the capital of the UK? |
|  |  | Early | The River Thames flows through which city? |
|  |  | Late | In which city does the River Thames flow? |
|  |  | Early | The big leaning tower is in which country? |
|  |  | Late | Which country is home to the big leaning tower? |
|  |  | Early | Which city, home to the Colosseum and Arch of Titus is located in Italy? |
|  |  | Late | Which Italian city is home to the Arch of Titus and Colosseum? |
|  |  | Early | A hexagon has how many sides? |
|  |  | Late | How many sides does a hexagon have? |
|  |  | Early | Which creature has a horn and appears in legends? |
|  |  | Late | Which creature appears in legends and has a horn? |
|  |  | Early | A pentagon has how many sides? |
|  |  | Late | How many sides does a pentagon have? |
|  |  | Early | Which country is home to the Berlin wall and is in Europe? |
|  |  | Late | Which country in Europe is home to the Berlin wall? |
|  |  | Early | Which household pet eats carrots and has two ears? |
|  |  | Late | Which household pet has two ears and eats carrots? |
|  |  | Early | An octagon has how many sides? |
|  |  | Late | How many sides does an octagon have? |
|  |  | Early | What object can be used to open locks and is also made of metal? |
|  |  | Late | What object is made of metal and can also be used to open locks? |
|  |  | Early | The circle on the Japanese flag, which is also known as circle of the sun, is what colour? |
|  |  | Late | What colour is the circle of the sun, which appears on the Japanese flag? |
|  |  | Early | Great Whites and Hammerheads, which live in the ocean, are what type of animal? |
|  |  | Late | What type of animal lives in the ocean and includes Great Whites and Hammerheads? |
|  |  | Early | What is the name of the fictional character who is Winnie the Pooh's donkey friend and was created by Disney? |
|  |  | Late | What is the name of the fictional character who was created by Disney and is Winnie the Pooh's donkey friend? |
|  |  | Early | What colour are emeralds, which are also gemstones? |
|  |  | Late | What colour is the gemstone known as an emerald? |
|  |  | Early | What word is the name of hot liquid that erupts from a volcano and begins with the letter L? |
|  |  | Late | What word begins with the letter L and is the name of hot liquid that erupts from a volcano? |
|  |  | Early | What food makes up nearly all of the diet of the animal known as the giant panda, that is also a mammal? |
|  |  | Late | What food makes up nearly all of the diet of the animal that is a mammal and also known as a giant panda? |
|  | Long | Early | Which date is Halloween and a time when people usually dress up? |
|  |  | Late | Which date do people usually dress up and celebrate Halloween? |
|  |  | Early | Which disney movie set on the sea stars Johnny Depp? |
|  |  | Late | Which disney movie starring Johnny Depp is set on the sea? |
|  |  | Early | Which platform, that appears in Harry Potter, can be found at Kings Cross station? |
|  |  | Late | Which platform can be found at Kings Cross station and appears in Harry Potter? |
|  |  | Early | Charlie Buckett is a character in which Johnny Depp movie? |
|  |  | Late | In which Johnny Depp movie does the character Charlie Buckett appear? |
|  |  | Early | Which Disney movie is about two dogs falling in love and was released in 1955? |
|  |  | Late | Which Disney movie, released in 1955, is about two dogs falling in love? |
|  |  | Early | What is the proper response to the phrase "see you later alligator" which is also a way of saying goodbye? |
|  |  | Late | What is the proper response to the phrase, which is also a way of saying goodbye, "see you later, alligator"? |
|  |  | Early | Which book, written by Lemony Snickett, has been made into a Netflix television series? |
|  |  | Late | Which book has been made into a Netflix television series and was written by Lemony Snickett? |
|  |  | Early | Which book series, written by J R R Tolekin, has also been made into films? |
|  |  | Late | Which book series has been made into a film and was written by J R R Tolkein? |
|  |  | Early | Which sculpture in New York is also located on an island? |
|  |  | Late | Which sculpture is located on an island in New York? |
|  |  | Early | Harry Potter's two best friends are called what? |
|  |  | Late | What are the full names of Harry Potter's two best friends? |
|  |  | Early | Arachnophobia is what? |
|  |  | Late | What is arachnophobia? |
|  |  | Early | Which television series, about a woman's prison, appears on Netflix? |
|  |  | Late | Which television series on Netflix is about a woman's prison? |
|  |  | Early | New Year’s Eve is on which date? |
|  |  | Late | What date is New Year’s Eve? |
|  |  | Early | Which scientist invented the telephone and was also born in Scotland? |
|  |  | Late | Which scientist, born in Scotland, invented the telephone? |
|  |  | Early | What happens to Pinocchio every time he tells a lie? |
|  |  | Late | Every time he tells a lie, what happens to Pinocchio? |
|  |  | Early | The man named Barack Obama, who also has two children, was the first African American to become what? |
|  |  | Late | The man who has two children and is also named Barack Obama was the first African-American to become what? |
|  |  | Early | What is the name of the book series written by George R R Martin that has also been made into a television series? |
|  |  | Late | What is the name of the book series, that has also been made into a television series, written by George R R Martin? |
|  |  | Early | The song Under the Sea is featured in which Disney movie? |
|  |  | Late | Which Disney movie features the song Under the Sea? |
|  |  | Early | What colours are the animals known as pandas that are also fluffy? |
|  |  | Late | What colours are the fluffy animals known as pandas? |
|  |  | Early | Which two toppings go on a Hawaiian pizza in addition to cheese and tomato? |
|  |  | Late | Which two toppings, along with cheese and tomato, usually go on a Hawaiian pizza? |
|  |  | Early | Which building, home to the president, is in Washington? |
|  |  | Late | Which building is in Washington and home to the president? |
|  |  | Early | Which play, written by William Shakespeare, is about two lovers? |
|  |  | Late | Which play is about two lovers and was written by William Shakespeare? |
|  |  | Early | Water boils at what temperature and can also be drank? |
|  |  | Late | Water, which can also be drank, boils at what temperature? |
| 2 & 3 | Short | Early | Which animal meows and also has four legs? |
|  |  | Late | Which animal has four legs and also meows? |
|  |  | Early | Which animal barks and is also a common household pet? |
|  |  | Late | Which animal is a common household pet and also barks? |
|  |  | Early | Which number indicates the number of sides a hexagon has and is even? |
|  |  | Late | Which number is even and indicates the number of sides a hexagon has? |
|  |  | Early | Which company is represented by a fruit and makes touchscreen phones? |
|  |  | Late | Which company makes touchscreen phones and is represented by a fruit? |
|  |  | Early | Which city is the capital of Scotland and is home to a castle? |
|  |  | Late | Which city is home to a castle and is the capital of Scotland? |
|  |  | Early | Which country is home to the pyramids and is located in Africa? |
|  |  | Late | Which country is located in Africa and is home to the pyramids? |
|  |  | Early | Which city is home to Buckingham Palace and is in the UK? |
|  |  | Late | Which city is in the UK and is home to Buckingham Palace? |
|  |  | Early | Which city is home to the Colleseum and is located in Italy? |
|  |  | Late | Which city is located in Italy and is home to the Colleseum? |
|  |  | Early | Which city is home to the big leaning tower and is located in a European country? |
|  |  | Late | Which city is located in a European country and is home to the big leaning tower? |
|  |  | Early | Which number indicates the number of sides a pentagon has and is less than twenty? |
|  |  | Late | Which number is less than twenty and indicates the number of sides a pentagon has? |
|  |  | Early | Which country is home to the Berlin wall and is in Europe? |
|  |  | Late | Which country is in Europe and is home to the Berlin wall? |
|  |  | Early | Which household pet eats carrots and has two ears? |
|  |  | Late | Which household pet has two ears and eats carrots? |
|  |  | Early | Which number indicates the number of sides an octagon has and is less than ten? |
|  |  | Late | Which number is less than ten and indicates the number of sides an octagon has? |
|  |  | Early | What object can be used to open locks and is made of metal? |
|  |  | Late | What object is made of metal and can be used to open locks? |
|  |  | Early | Which city is the capital of the The Netherlands and also begins with the letter 'A'? |
|  |  | Late | Which city begins with the letter 'A' and is also the capital of the The Netherlands? |
|  |  | Early | Which country is home to the Sydney Opera House and begins with the letter "A"? |
|  |  | Late | Which country begins with the letter "A" and is home to the Sydney Opera House? |
|  |  | Early | Which type of animal includes the species Hammerheads and lives in the ocean? |
|  |  | Late | Which type of animal lives in the ocean and includes the species Hammerheads? |
|  |  | Early | What is the name of the fictional character who is Winnie the Pooh's donkey friend and was created by a British author? |
|  |  | Late | What is the name of the fictional character who was created by a British author and is Winnie the Pooh's donkey friend? |
|  |  | Early | What is the colour of an emerald and is a colour that appears in the rainbow? |
|  |  | Late | What is a colour that appears in the rainbow and is the colour of an emerald? |
|  |  | Early | What word is the name of hot liquid that erupts from a volcano and begins with the letter L? |
|  |  | Late | What word begins with the letter L and is the name of hot liquid that erupts from a volcano? |
|  |  | Early | What food is eaten by a giant panda and can be grown? |
|  |  | Late | What food can be grown and is eaten by a giant panda? |
|  |  | Early | Which animal has eight tentacles and lives in the sea? |
|  |  | Late | Which animal lives in the sea and has eight tentacles? |
|  |  | Early | What is the name of the fairy who appears in Peter Pan and wears green? |
|  |  | Late | What is the name of the fairy who wears green and appears in Peter Pan? |
|  |  | Early | What word is a measurement of temperature in degrees and begins with the letter C? |
|  |  | Late | What word begins with the letter C and is a measurement of temperature in degrees? |
|  |  | Early | Which character wore a glass slipper and appeared in a Disney movie? |
|  |  | Late | Which character appeared in a Disney movie and wore a glass slipper? |
|  |  | Early | Which sport involves kicking a ball and is played by a team? |
|  |  | Late | Which sport is played by a team and involves kicking a ball? |
|  |  | Early | Which cartoon cat is known for eating lasagne and appeared on television? |
|  |  | Late | Which cartoon cat appeared on television and is known for eating lasagne? |
|  |  | Early | Which sport involves a shuttlecock and is played by two or four people? |
|  |  | Late | Which sport is played by two or four people and involves a shuttlecock? |
|  |  | Early | Which swimming stroke is named after an insect and requires both your arms and legs? |
|  |  | Late | Which swimming stroke requires both your arms and legs and is named after an insect? |
|  |  | Early | Which object is used by witches to fly and can be found in a house? |
|  |  | Late | Which object can be found in a house and is used by witches to fly? |
|  |  | Early | Which boat hit an iceberg and carried lots of people? |
|  |  | Late | Which boat carried lots of people and hit an iceberg? |
|  |  | Early | Which insect is red with black spots and can fly? |
|  |  | Late | Which insect can fly and is red with black spots? |
|  |  | Early | Which city is the capital of China and is home to a big airport? |
|  |  | Late | Which city is home to a big airport and is the capital of China? |
|  |  | Early | Which fruit is used to make wine and can be eaten? |
|  |  | Late | Which fruit can be eaten and is used to make wine? |
|  |  | Early | What type of dog is found on racetracks and can be brown? |
|  |  | Late | What type of dog can be brown and is found on racetracks? |
|  | Long | Early | Which Disney movie is set on the sea and stars Johnny Depp? |
|  |  | Late | Which Disney movie stars Johnny Depp and is set on the sea? |
|  |  | Early | Which platform appears in Harry Potter and can be found at Kings Cross station? |
|  |  | Late | Which platform can be found at Kings Cross station and appears in Harry Potter? |
|  |  | Early | Which movie stars the character Charlie Buckett and is about a factory? |
|  |  | Late | Which movie is about a factory and stars the character Charlie Buckett? |
|  |  | Early | Which Disney movie is about two dogs falling in love and was released in 1955? |
|  |  | Late | Which Disney movie was released in 1955 and is about two dogs falling in love? |
|  |  | Early | What is the proper response to the phrase "see you later alligator" which is also a way of saying goodbye? |
|  |  | Late | What is the proper response to the phrase, which is also a way of saying goodbye, "see you later, alligator"? |
|  |  | Early | Which book was written by Lemony Snickett and has been made into a Netflix television series? |
|  |  | Late | Which book has been made into a Netflix television series and was written by Lemony Snickett? |
|  |  | Early | Which statue is in New York and is located on an island? |
|  |  | Late | Which statue is located on an island and is in New York? |
|  |  | Early | What are the names of the two characters that are Harry Potter's best friends and appear in both the books and the films? |
|  |  | Late | What are the names of the two characters that appear in both the books and the films and are Harry Potter's best friends? |
|  |  | Early | Which television series is about a woman's prison and appears on Netflix? |
|  |  | Late | Which television series appears on Netflix and is about a woman's prison? |
|  |  | Early | Which date is known as New Year's Eve and is celebrated around the world? |
|  |  | Late | Which date is celebrated around the world and is known as New Year's Eve? |
|  |  | Early | What happens to Pinocchio when he tells a lie in the famous Disney story? |
|  |  | Late | What happens in the famous Disney story to Pinocchio when he tells a lie? |
|  |  | Early | Which Disney movie featured the song Under the Sea and appeared in most cinemas? |
|  |  | Late | Which Disney movie appeared in most cinemas and featured the song Under the Sea? |
|  |  | Early | On what date do people celebrate Halloween and usually dress up? |
|  |  | Late | On what date do people usually dress up and celebrate Halloween? |
|  |  | Early | What are the colours of pandas and are the two colours that do not appear in the rainbow? |
|  |  | Late | What are the two colours that do not appear in the rainbow and are the colours of pandas? |
|  |  | Early | Which two toppings usually go on a Hawaiian pizza in addition to cheese and tomato? |
|  |  | Late | Which two toppings, in addition to cheese and tomato, usually go on a Hawaiian pizza? |
|  |  | Early | Which building is home to the president and is in America? |
|  |  | Late | Which building is in America and is home to the president? |
|  |  | Early | Which temperature does water boil at on a thermometer? |
|  |  | Late | Which temperature on a thermometer does water boil at? |
|  |  | Early | Which date is when Santa Claus visits and is a national holiday? |
|  |  | Late | Which date is a national holiday and is when Santa Claus visits? |
|  |  | Early | Which address is home to the prime minister and is in London? |
|  |  | Late | Which address is in London and is home to the prime minister? |
|  |  | Early | Which artist painted the Mona Lisa and was Italian? |
|  |  | Late | Which artist was Italian and painted the Mona Lisa? |
|  |  | Early | Which location is home to Santa Claus and is also cold? |
|  |  | Late | Which location is cold and is also home to Santa Claus? |
|  |  | Early | Which tourist attraction is in Paris and is very tall? |
|  |  | Late | Which tourist attraction is very tall and is in Paris? |
|  |  | Early | What is the name of the novel that was written by Harper Lee and was published in 1960? |
|  |  | Late | What is the name of the novel that was published in 1960 and was written by Harper Lee? |
|  |  | Early | Which movie features the character Quasimodo and was created by Disney? |
|  |  | Late | Which movie was created by Disney and features the character Quasiomodo? |
|  |  | Early | What was the name of the English king who had six wives and who was a famous monarch? |
|  |  | Late | What was the name of the English King who was a famous monarch and who had six wives? |
|  |  | Early | Which movie features the character Simba and is animated? |
|  |  | Late | Which movie is animated and features the character Simba? |
|  |  | Early | What object could make Harry Potter invisible and appeared in the famous movies? |
|  |  | Late | What object appeared in the famous movies and could make Harry Potter invisible? |
|  |  | Early | Which Star Wars character is known for the line "I am your father" and appears in the famous movies? |
|  |  | Late | Which Star Wars character appears in the famous movies and is known for the line "I am your father"? |
|  |  | Early | Who wrote Romeo and Juliet and was a well known writer? |
|  |  | Late | Who was a well known writer and wrote Romeo and Juliet? |
|  |  | Early | What was the name of the egg that had a great fall who was also a character in the popular children's story? |
|  |  | Late | What was the name of the character in the popular children's story who was also an egg that had a great fall? |
|  |  | Early | What is the name of the author who wrote the Harry Potter series and is famous? |
|  |  | Late | What is the name of the author who is famous and wrote the Harry Potter series? |
|  |  | Early | Which Disney movie featured the character Belle and appeared in most cinemas? |
|  |  | Late | Which Disney movie appeared in most cinemas and featured the character Belle? |
|  |  | Early | Which Disney movie featured the villain Cruella De Vil and appeared in most cinemas? |
|  |  | Late | Which Disney movie appeared in most cinemas and featured the villain Cruella De Vil? |
|  |  | Early | Who lost her sheep in the nursery rhyme often told to children? |
|  |  | Late | In the nursery rhyme often told to children, who lost her sheep? |
|  |  | Early | Which famous building was almost blown up by Guy Fawkes in London? |
|  |  | Late | Which famous building in London was almost blown up by Guy Fawkes? |

Table A2. Full model output for the linear mixed effects model analysis for all experiments. The lmer formula for all experiments was: Answer times ~ Critical Content * Answer Length + (1 + Critical Content + Answer Length || Participant) + (1 + Critical Content || Item). RE var = Random effects variance; (p) stands for random effects by participants; (i) stands for random effects by items. All predictors are defined in the Data Analysis section for each experiment.

|  | Experiment 1 | | | | Experiment 2 | | | | Experiment 3 | | | |
| --- | --- | --- | --- | --- | --- | --- | --- | --- | --- | --- | --- | --- |
|  | *b* | SE | *t* | RE variance | *b* | SE | *t* | RE variance | *b* | SE | *t* | RE variance |
| Intercept | 406.94 | 71.27 | 5.71 | (p) 242576; (i) 100288 | 149.88 | 69.37 | 2.16 | (p) 207911; (i) 88679 | 844.88 | 44.98 | 18.79 | (p) 78008; (i) 42482 |
| Critical Content | -608.31 | 76.90 | -7.91 | (p) 76534;  (i) 186362 | -735.68 | 80.00 | -9.10 | (p) 179582; (i) 190582 | -180.91 | 31.99 | -5.66 | (p) 5559; (i) 20932 |
| Answer Length | -234.13 | 100.48 | -2.33 | (p) 22628 | -100.34 | 79.16 | -1.27 | (p) 1618 | -29.27 | 59.05 | -0.50 | (p) 14960 |
| Critical Content * Answer Length | 40.13 | 142.37 | 0.28 | - | 236.09 | 117.93 | 2.00 | - | -8.17 | 60.93 | -0.13 | - |
| Question Duration | -205.01 | 48.75 | -4.21 | - | -194.43 | 39.22 | -4.95 | - | 3.10 | 27.58 | 0.11 | - |
